# Lung function in Lolland‐Falster Health Study (LOFUS)

**DOI:** 10.1111/crj.13536

**Published:** 2022-09-02

**Authors:** Katja Kemp Jacobsen, Randi Jepsen, Uffe Bodtger, Knud Rasmussen, Gry St‐Martin

**Affiliations:** ^1^ Department of Technology, Faculty of Health and Technology University College Copenhagen Copenhagen Denmark; ^2^ Centre for Epidemiological Research Nykøbing Falster Hospital Nykøbing Falster Denmark; ^3^ Pulmonary Research Unit Region Zealand (PLUZ), Department of Respiratory Medicine Naestved Hospital Naestved Denmark; ^4^ Department of Regional Health Research University of Southern Denmark Odense Denmark; ^5^ Department of Internal Medicine Zealand University Hospital Roskilde Roskilde Denmark; ^6^ Data and Development Support Region Zealand Sorø Denmark

**Keywords:** COPD, COPD prevalence, criteria, FEV_1_/FVC ratio, lower limit of normal, lung function, spirometry

## Abstract

**Background:**

COPD prevalence in Denmark is estimated at 18% based on data from urban populations. However, studies suggest that using the clinical cut‐off for airway obstruction in population studies may overestimate prevalence. The present study aims to compare estimated prevalence of airway obstruction using different cut‐offs and to present lung function data from the Lolland‐Falster Health Study, set in a rural‐provincial area.

**Methods:**

Descriptive analysis of participant characteristics and self‐reported respiratory disease and of spirometry results in the total population and in subgroups defined by these characteristics. Airway obstruction was assessed using previously published Danish reference values and defined according to either FEV_1_/FVC below lower limit of normal (LLN) 5% (as in clinical diagnosis) or 2.5% (suggested for population studies), or as FEV_1_/FVC < 70%.

**Results:**

Using either FEV_1_/FVC < 70% or LLN 5% cut‐off, 19.0% of LOFUS participants aged 35 years or older had spirometry, suggesting airway obstruction. By the LLN 2.5% criterion, the proportion was considerably lower, 12.2%. The prevalence of airway obstruction was higher among current smokers, in participants with short education or reporting low leisure‐time physical activity and in those with known respiratory disease. Approximately 40% of participants reporting known respiratory disease had normal spirometry, and 8.7% without known respiratory disease had airway obstruction.

**Conclusion:**

Prevalence of airway obstruction in this rural population was comparable to previous estimates from urban Danish population studies. The choice of cut‐off impacts the estimated prevalence, and using the FEV_1_/FVC cut‐off may overestimate prevalence. However, many participants with known respiratory disease had normal spirometry in this health study.

## Graphical abstract

Prevalence of airway obstruction from population studies are based on spirometry cut‐off intended for clinical use. This study highlights how different cut‐off impact the estimate. Using the lower limit of normal (LLN) 2.5% cut‐off, which may be preferable for population studies, prevalence of airway obstruction was considerably lower compared to estimates using LLN 5% (as in clinical diagnosis) and FEV_1_/FVC.

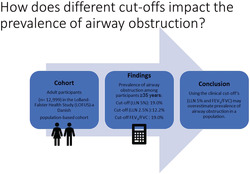

## 1 BACKGROUND

Worldwide, lung disease is a major cause of morbidity and mortality.^1^ Lung function declines with age,^2^ and smoking and environmental pollution can cause lung disease, accelerated loss of lung function and premature death.^3^, ^4^, ^5^, ^6^

In Denmark, prevalence of chronic obstructive pulmonary disease (COPD) has been estimated at 17.5% of adults aged 35 years or older,^7^ with estimated 2.0% having severe COPD.^7^ This estimate derived from a large urban population study using a spirometry cut‐off intended for clinical diagnosis, that is, in a clinical setting with suspicion of airway disease. This may lead to overestimation of COPD prevalence.^8^, ^9^ In addition, the choice of spirometry criterion to most correctly detect airway obstruction has been debated in recent decades. Detection of reduced ratio between forced expiratory volume 1 s (FEV_1_) and forced vital capacity (FVC) indicates airway obstruction, and while a fixed ratio criterion of FEV_1_/FVC < 0.70 is often used, studies show that FEV_1_/FVC ratio below a lower limit of normal (LLN) may be more suitable.^8^, ^10^

Lolland‐Falster is a mixed rural‐provincial area of 103 000 inhabitants, situated on two main and several small islands in the southern part of Denmark, a small Scandinavian, high‐income country covering 43 000 km^2^ and a population of 5.8 million. Although the Danish population is genetically relatively homogeneous, population health varies across regions of the country, with life expectancy in Lolland‐Falster hree 3 years below the national average and 5 years below the municipalities with highest life expectancy.^11^, ^12^ The region scores worse than the national average on several health indicators, including diabetes prevalence, obesity, smoking and COPD.^13^

The Lolland‐Falster Health Study (LOFUS) is a population‐based, prospective cohort study designed to investigate determinants of population health in this area.^14^ In this paper, we report LOFUS data on lung function measurement with spirometry as well as anthropometric data and questionnaire‐based information on smoking and other risk factors. The aim is to describe the spirometry measurements and results in adults participating in LOFUS and compare it to similar findings from urban Danish population. In addition, we explore how different spirometric criteria affect the estimated prevalence of airway obstruction.

## 2 METHODS AND DATA

### 2.1 LOFUS

LOFUS is a household‐based study where households of randomly selected persons aged 18 and above were invited to participate.^14^ The data collection encompassed self‐administered, age‐specific questionnaires on social, mental and physical health and lifestyle factors; anthropometric and physiological measurements undertaken in the study clinic; and collection of biological samples. The data collection started in February 2016 and ended February 2020.

### 2.2 Spirometry

Lung function was measured by trained healthcare professionals using the MicroLoop Handheld Spirometer™ and SpiroUSB™ with Spirometry PC Software (CareFusion Corp., USA). Sex, height and ethnic origin (Caucasian or Asian) were entered into the software, and the spirometry was performed in a standing position (if possible) with the use of a nose clip. There were no restrictions on behaviour or medication prior to the measurement, and bronchodilator was not administered prior to spirometry. The spirometer was calibrated once a week. Three sets of values were obtained for FEV_1_,FVC, and as a criterion for correct performance, the two highest measurements might differ only by ≤0.150 L, and all measurements should be defined as ‘Good blow’ or ‘Short blow’ by the spirometer. The highest value of both FEV_1_ and FVC for each participant was used in the analysis, and the ratio of FEV_1_ to FVC (FEV_1_/FVC) was calculated for each participant.

### 2.3 Other variables

We examined a number of potential determinants of lung function selected from the literature. From the measurements in the clinic, we used information on height (cm), waist circumference (cm) and weight (kg). Body mass index (BMI) was calculated as weight/height^2^ and categorized into underweight (<18.5), normal weight (18.5–24.9), overweight (25.0–29.9) and obese (≥30.0).^15^ Waist circumference was grouped into normal or large (with limits of >94 cm for men and >80 cm for women).^16^

From the questionnaire, we used self‐reported smoking data categorized as current (daily or sometimes), former and never. Number of pack‐years was calculated for current smokers, with 1 pack‐year corresponding to 20 cigarettes or equivalent per day for 1 year. School education was divided into ≤7 years; 8–9 years; 10–11 years; graduated high school; under education; and other. Vocational education was divided into primary school only; semiskilled worker, for example, truck driver; vocational training, for example, hairdresser; short higher education, for example, laboratory worker; middle higher education, for example, school teacher; long higher education, for example, master's degree or equivalent; and other education. Physical activity was based on the following question: ‘How would you characterize your leisure time physical activity within the last year?’ and classified as sedentary, moderate, heavy activity or heavy activity at competition level.^17^

Self‐reported prevalent morbidity was based on the following question from the LOFUS study questionnaire: ‘Do you suffer from any of the following diseases?’ Participants were asked to mark either yes or no for each of the following categories: ‘asthma’, ‘chronic bronchitis, hyperinflated lungs, chronic obstructive pulmonary disease (COPD), or emphysema’, ‘heart attack’, ‘atherosclerosis in the heart’, ‘angina’, ‘hypertension’, ‘diabetes’ and ‘cancer’. Information was merged with self‐reported daily medication use and categorized as asthma, other respiratory disease (including COPD, chronic bronchitis and emphysema), allergy, hypertension, diabetes, cancer or ischaemic heart disease.

### 2.4 Data analysis

We included all 16 142 adults (i.e. aged 18 years and above) from the LOFUS study in the descriptive analyses of participant characteristics. Mean and standard deviation (SD) were calculated for characteristics of all participants. Before analysis, we checked for differences between those with successful and not successful spirometry using Kruskal–Wallis test and Pearson's *X* ^2^ test.

**FIGURE 1:**
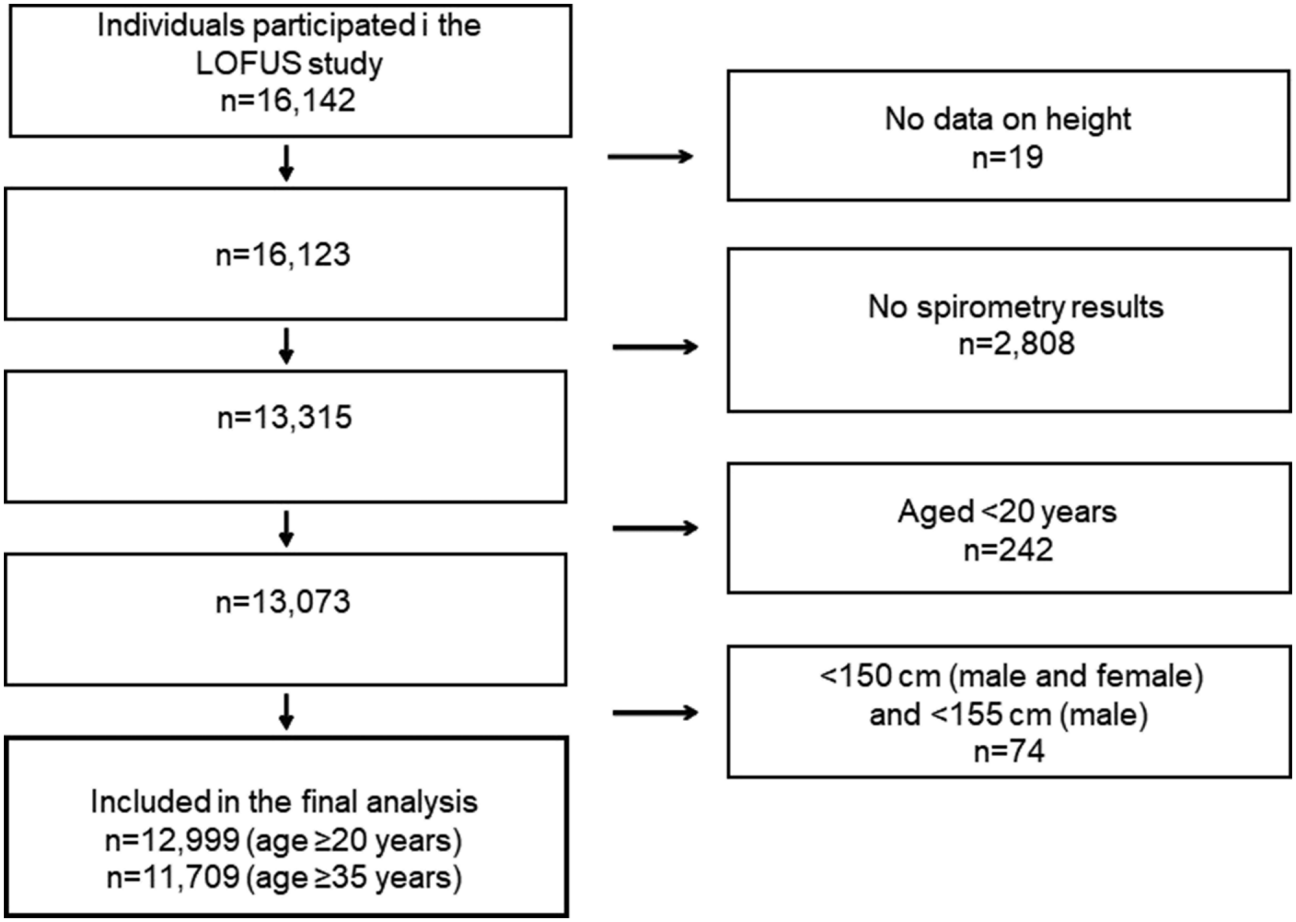
Flowchart of the study population among 16 142 individuals aged ≥18 years in the LOFUS study

We then compared spirometry results with the reference values stated for the Danish normal population by Løkke et al.^18^ For this part of the analysis, we excluded participants with age <20 years, height <150 cm (male and female) or <155 cm (male) to match the population in the reference material (Figure 1). We considered a reduction in the ratio FEV_1_/FVC to be indicative of airway obstruction and compared three different cut‐offs: FEV_1_/FVC < 70%, FEV_1_/FVC (LLN 5%) and LLN 2.5%. LLN 5% was stated for the Danish normal population by Løkke et al.^18^ The LLN 2.5% was not stated in the study but calculated by subtracting 1.96 × residual SD from the predicted mean (assuming a Gaussian distribution of the residuals). For each participant, we determined if FEV_1_, FVC and FEV_1_/FVC were above or below the LLN 5% and the LLN 2.5% corresponding to that participant's age, sex, and height. We then calculated the proportions with FEV_1_, FVC and FEV_1_/FVC below LLN and the proportion with FEV_1_/FVC < 70%. The proportion with FEV_1_, FVC and FEV_1_/FVC below LLN and the proportion with FEV_1_/FVC < 70% in subgroups of participants defined by sex, age and other variables were tabulated.

Analyses were carried out for all participants ≥20 years. As COPD is usually diagnosed in middle‐aged or older adults, we also carried out analysis without the younger age groups. We chose 35 years as cut‐off, because this was used in a previous Danish prevalence study^7^ (Figure 1). For the next part of the analysis, we excluded participants <35 years, leaving 11 709 participants for analysis. The proportion with FEV_1_/FVC below LLN 2.5 and 5.0% and the proportion with FEV_1_/FVC < 70% in subgroups of participants were tabulated. Logistic regression was used to evaluate variables associated with airway obstruction defined as the proportion with FEV_1_/FVC below LLN 2.5 and 5.0% and the proportion with FEV_1_/FVC < 70%. Analyses were performed using STATA/SE 15.1.

### 2.5 Ethics

Informed written consent was obtained from all LOFUS participants. The LOFUS study was approved by the Region Zealand's Ethical Committee on Health Research (SJ‐421) and the Danish Data Protection Agency (REG‐024‐2015). LOFUS is registered in Clinical Trials (NCT02482896).

## 3 RESULTS

### 3.1 Participant characteristics

Spirometry measurements were tried for all 16 142 participants, 19 participants were excluded due to missing height, and of the 16 123 participants, 13 315 (82.5%) completed three acceptable measurements (Figure 1). Participants with unsuccessful spirometry were more likely to be men, old, overweight, sedentary, having large waist circumference, low school/vocational education, hypertension or ischaemic heart disease and less likely having asthma and other respiratory disease.

The population with successful spirometry was on average 59.0 years with 54.2% being women. The majority were overweight (63.4%), had education below high school graduation (62.5%), reported moderate physical activity (61.3%) or were former or current smokers (51.8%), and 26.8% reported to have hypertension (Table S1).

### 3.2 Respiratory disease

The participants with other respiratory disease (*n* = 1028) was on average 61.2 years with 56.4% being women (Table S2). 75.1% reported also to have asthma. Compared to the total group with successful spirometry (Table S1), the participants with self‐reported respiratory disease was more likely to be older, have higher waist circumference, lower school education, more sedentary activity, be current daily smokers with more pack‐years and have asthma, allergy, hypertension, diabetes, cancer and ischaemic heart disease.

### 3.3 Spirometry results

Table 1 shows proportions of participants who met the three different criteria for airway obstruction, by age categories, anthropometric data, educational status and smoking status in participants aged 35 years or older. Overall 12.2% of participants had FEV_1_/FVC below the LLN 2.5% cut‐off, 19.0% below the LLN 5%, and 19.0% below 70%, but with variation by age group. Up until age 49, the proportions meeting the FEV_1_/FVC < 70% and the LLN 2.5% criteria were similar, but with increasing age, the proportion with FEV_1_/FVC < 70% increased more than the proportion meeting the LLN criteria. As a result, the difference between the proportions of FEV_1_/FVC < 70% and FEV_1_/FVC < LLN 2.5% increased in the oldest age groups.

**TABLE 1:** Proportion of participants with FEV1/FVC < LLN 2.5%, LLN 5% and <70% among all aged ≥35 years (*n* = 11 709) with successful spirometry in the LOFUS study

	Number	Proportion with FEV_1_/FVC < LLN 2.5%(%)	Proportion with FEV_1_/FVC < LLN 5.0%(%)	Proportion with FEV_1_/FVC < 70%(%)
Total, age ≥35 years	11 709	12.2	19.0	19.0
Sex				
Male	5390	12.8	19.4	19.2
Female	6319	11.6	18.7	18.8
Age				
35–39	687	8.2	14.9	6.4
40–49	2071	8.4	14.6	8.2
50–59	2954	11.3	18.9	14.6
60–69	3219	14.2	21.1	22.5
70–79	2280	14.7	21.0	29.2
80+	498	14.5	21.7	37.2
BMI (kg/m^2^)				
BMI <18.5	115	27.8	43.5	42.6
BMI 18.5–25	3860	14.5	22.4	22.3
BMI 25.1–30	4525	11.5	18.1	18.0
BMI >30	3000	9.3	14.9	14.6
Waist circumference (cm)				
<94 cm/80 cm	2692	13.6	22.4	19.8
≥94 cm/80 cm	8943	11.7	18.2	18.7
School education				
≤7 years	1171	16.8	24.3	30.8
8–9 years	2233	14.5	21.4	23.0
10–11 years	3833	11.0	18.0	17.2
Graduated high school	2959	9.7	17.1	14.4
Under education	17	17.7	23.5	17.7
Other	803	12.2	17.2	15.6
Vocational education				
No education except from primary school	1015	15.5	22.6	24.1
Smaller courses (e.g. semiskilled worker)	666	15.9	25.2	24.2
Vocational training	4803	11.8	18.7	18.4
Short higher education	995	10.9	16.0	17.2
Middle higher education	2532	10.4	17.4	16.5
Long higher education	507	11.4	19.5	17.2
Other	616	15.3	21.8	24.5
Physical activity last year				
Sedentary activity	1268	17.2	24.5	25.0
Moderate activity	7016	12.1	19.1	19.1
Heavy activity	2699	9.9	16.7	16.4
Heavy activity, at competition level	155	9.0	13.6	10.3
Smoking				
Current daily smokers	1810	25.6	36.0	33.2
Current sometimes smokers	273	16.1	26.0	23.1
Former‐smokers	4237	14.0	21.2	22.3
Never‐smokers	4862	5.3	10.6	10.7
Cumulative smoking (pack‐years)				
>0–20	854	19.0	29.7	26.1
20–40	706	26.9	35.8	33.0
40–60	265	37.0	49.8	47.6
>60	88	36.4	53.4	51.1
Chronic diseases				
Asthma	876	41.3	50.9	49.2
Other respiratory disease^a^	951	46.7	56.5	57.6
Allergy	2036	14.6	22.3	21.0
Hypertension	3515	12.6	19.5	22.7
Diabetes	662	11.8	19.9	21.2
Cancer	478	18.2	25.3	28.7
Ischaemic heart disease	1309	15.2	22.8	27.3

*Notes*: LLN is defined by normal values in a Danish population.^17^ Values are number (frequencies) for categorical values. *P*‐values were calculated with Pearson's *X* ^2^ test for categorical values.

^a^ Other respiratory disease includes COPD, chronic bronchitis and emphysema.

As expected, proportions were higher in smokers, especially current smokers and those with most pack‐years, and among participants reporting other respiratory disease. In addition, spirometry results below expected were more common among those with fewer years of schooling and those reporting sedentary or moderate physical activity. The proportion of participants with FEV_1_/FVC < 70% and FEV_1_/FVC LLN 2.5% was highest among those with BMI < 18.5 (42.6% and 27.8%, respectively), low school education (30.8% and 16.8%), sedentary activity (25.0% and 17.2%), current daily smoking (33.2% and 25.6%), asthma (49.2% and 41.3%) and other respiratory disease (57.6% and 46.7%).

The highest proportion of participants with FEV_1_/FVC < 70% was observed among current daily smokers (33.2%) and among those with known asthma (49.2%) or other respiratory disease (57.6%). The corresponding values for FEV_1_/FVC below LLN 2.5 were 25.6%, 41.3% and 46.7%, respectively.

Table 2 shows results of univariable and multivariable analyses for proportion with airway obstruction using the different criteria. The strongest association was seen in daily smokers, followed by sometimes smokers and former smokers. Increasing number of pack‐years was also strongly associated with airway obstruction by either criterion. Airway obstruction was associated with increasing age for the fixed ratio criterion but not for LLN. BMI < 18.5 was associated with airway obstruction, as was sedentary lifestyle. Asthma and other respiratory disease were associated with airway obstruction, while other chronic diseases were not. No clear association was found for educational level or waist circumference.

**TABLE 2:** Association of characteristics and FEV1/FVC < LLN 2.5%, FEV1/FVC < LLN 5% and proportion with FEV_1_/FVC < 70% among all aged ≥35 years (*n* = 11 709) with successful spirometry in the LOFUS study according to age

	FEV_1_/FVC < LLN 2.5%		FEV_1_/FVC < LLN 5.0%		FEV_1_/FVC < 70%	
	Univariable model	Multivariable model^a^ *n* = 10 739	Univariable model	Multivariable model^a^ *n* = 10 739	Univariable model	Multivariable model^a^ *n* = 10 739
Sex, female	0.90(0.80–1.00)	0.78(0.64–0.95)	0.96(0.87–1.05)	0.81(0.69–0.95)	0.98(0.89–1.07)	1.41(1.19–1.66)
Age (years)						
Age, per year increase	1.02(1.01–1.02)		1.01(1.00–1.02)		1.05(1.05–1.05)	
35–39	1.0	1.0	1.0	1.0	1.0	1.0
40–49	1.03(0.75–1.40)	0.94(0.66–1.33)	0.98(0.77–1.25)	0.98(0.75–1.29)	1.30(0.92–1.83)	1.28(0.87–1.88)
50–59	1.43(1.06–1.92)	1.20(0.86–1.68)	1.33(1.06–1.68)	1.21(0.93–1.58)	2.50(1.81–3.45)	2.49(1.73–3.58)
60–69	1.86(1.40–2.50)	1.44(1.03–2.03)	1.54(1.22–1.93)	1.25(0.96–1.63)	4.24(3.10–5.83)	4.37(3.04–6.28)
70–79	1.93(1.44–2.60)	1.57(1.10–2.25)	1.52(1.20–1.92)	1.28(0.96–1.70)	6.03(4.39–8.29)	7.31(5.02–10.64)
80+	1.90(1.32–2.76)	1.26(0.78–2.02)	1.59(1.18–2.14)	1.17(0.80–1.71)	8.64(6.05–12.32)	10.81(7.02–16.6)
Height (cm), pr cm increase	0.99(0.99–1.00)	0.99(0.98–1.00)	0.99(0.99–1.00)	0.99(0.98–1.00)	0.99(0.99–1.00)	1.02(1.01–1.03)
BMI (kg/m^2^)						
BMI <18.5	2.27(1.50–3.55)	1.56(0.94–2.58)	2.66(1.82–3.88)	2.03(1.31–3.16)	2.58(1.77–3.77)	2.18(1.38–3.44)
BMI 18.5–25	1.0	1.0	1.0	1.0	1.0	1.0
BMI 25.1–30	0.77(0.67–0.87)	0.70(0.59–0.83)	0.76(0.69–0.85)	0.70(0.60–0.80)	0.77(0.69–0.85)	0.69(0.60–0.80)
BMI >30	0.61(0.52–0.71)	0.52(0.42–0.64)	0.60(0.53–0.69)	0.52(0.44–0.61)	0.59(0.52–0.67)	0.53(0.45–0.63)
Waist circumference (cm)						
<94 cm/80 cm	1.0	1.0	1.0	1.0	1.0	1.0
≥94 cm/80 cm	0.84(0.74–0.96)	1.01(0.84–1.22)	0.82(0.74–0.91)	1.07(0.91–1.25)	0.93(0.84–1.04)	0.99(0.85–1.17)
School education						
≤7 years	1.0	1.0	1.0	1.0	1.0	1.0
8–9 years	0.84(0.69–1.02)	0.90(0.71–1.14)	0.84(0.71–1.00)	0.86(0.70–1.06)	0.67(0.57–0.78)	0.95(0.79–1.16)
10–11 years	0.61(0.51–0.74)	0.83(0.65–1.06)	0.68(0.59–0.80)	0.83(0.68–1.02)	0.47(0.40–0.54)	0.84(0.69–1.02)
Graduated high school	0.53(0.44–0.65)	0.87(0.65–1.15)	0.64(0.54–0.75)	0.88(0.70–1.12)	0.38(0.32–0.44)	0.86(0.68–1.09)
Under education	1.05(0.30–3.72)	1.25(0.31–5.08)	0.96(0.31–2.96)	0.98(0.28–3.42)	0.48(0.13–1.68)	1.24(0.27–5.65)
Other	0.69(0.53–0.89)	0.91(0.65–1.27)	0.65(0.51–0.81)	0.75(0.56–0.99)	0.41(0.33–0.52)	0.80(0.60–1.07)
Vocational education						
No education except from primary school	1.00	1.00	1.00	1.00	1.00	1.00
Smaller courses (e.g. semiskilled worker)	1.03(0.79–1.35)	1.02(0.75–1.40)	1.16(0.92–1.46)	1.19(0.92–1.54)	1.00(0.80–1.26)	1.15(0.88–1.50)
Vocational training	0.73(0.60–0.88)	0.92(0.74–1.16)	0.79(0.67–0.93)	0.98(0.81–1.19)	0.71(0.60–0.83)	0.97(0.80–1.18)
Short higher education	0.67(0.51–0.87)	1.03(0.75–1.39)	0.65(0.52–0.82)	0.92(0.71–1.20)	0.65(0.52–0.81)	1.06(0.82–1.37)
Middle higher education	0.63(0.51–0.78)	0.93(0.71–1.22)	0.72(0.60–0.86)	1.00(0.80–1.25)	0.62(0.52–0.74)	1.01(0.81–1.27)
Long higher education	0.71(0.51–0.97)	1.05(0.70–1.56)	0.83(0.64–1.08)	1.19(0.86–1.63)	0.65(0.50–0.85)	1.00(0.71–1.40)
Other	0.98(0.75–1.30)	1.12(0.81–1.54)	0.95(0.75–1.21)	1.08(0.82–1.42)	1.02(0.81–1.29)	1.17(0.89–1.54)
Physical activity last year						
Sedentary activity	1.00	1.00	1.00	1.00	1.00	1.00
Moderate activity	0.66(0.57–0.78)	0.78(0.65–0.95)	0.73(0.63–0.84)	0.83(0.70–0.98)	0.67(0.58–0.77)	0.80(0.68–0.95)
Heavy activity	0.53(0.44–0.64)	0.70(0.55–0.88)	0.62(0.52–0.73)	0.77(0.64–0.94)	0.56(0.48–0.66)	0.81(0.66–0.99)
Heavy activity, at competition level	0.48(0.27–0.84)	0.62(0.32–1.21)	0.48(0.30–0.78)	0.61(0.35–1.04)	0.35(0.20–0.60)	0.55(0.30–1.00)
Smoking						
Current daily smokers	6.13(5.20–7.22)	5.38(4.48–6.45)	4.76(4.17–5.44)	4.17(3.60–4.82)	4.15(3.63–4.74)	4.28(3.67–4.99)
Current sometimes smokers	3.41(2.41–4.83)	3.26(2.23–4.75)	2.97(2.23–3.96)	2.80(2.05–3.80)	2.62(1.93–3.57)	2.85(2.04–3.98)
Former‐smokers	2.89(2.48–3.36)	2.46(2.09–2.90)	2.27(2.02–2.56)	2.02(1.78–2.29)	2.36(2.10–2.66)	2.00(1.76–2.28)
Never‐smokers	1.00	1.00	1.00	1.00	1.00	1.00
Cumulative smoking (pack‐years)^a^						
0	1.00	NA	1.00	NA	1.00	NA
>0–20	4.16(3.37–5.14)	NA	3.58(3.01–4.26)	NA	2.95(4.47–3.52)	NA
20–40	6.54(5.31–8.06)	NA	4.72(3.95–5.65)	NA	4.11(3.43–4.93)	NA
40–60	10.40(8.89–13.8)	NA	8.40(6.49–10.8)	NA	7.57(5.85–9.79)	NA
>60	10.16(6.46–16.00)	NA	9.70(6.32–14.9)	NA	8.74(5.69–13.40)	NA
Chronic diseases						
Asthma	6.47(5.57–7.50)	2.18(1.66–2.86)	5.26(4.57–6.07)	1.88(1.46–2.42)	4.89(4.25–5.64)	1.57(1.20–2.05)
Other respiratory disease^b^	8.72(7.55–10.06)	4.28(3.32–5.51)	6.95(6.06–7.99)	3.77(2.98–4.78)	6.95(6.07–7.99)	4.37(3.40–5.61)
Allergy	1.30(1.13–1.49)	0.96(0.81–1.13)	1.27(1.13–1.43)	0.98(0.85–1.12)	1.17(1.04–1.31)	1.00(0.87–1.15)
Hypertension	1.06(0.94–1.20)	0.90(0.77–1.05)	1.05(0.95–1.16)	0.93(0.81–1.06)	1.40(1.27–1.54)	0.99(0.87–1.12)
Diabetes	0.96(0.75–1.23)	0.74(0.56–1.00)	1.06(0.87–1.29)	0.90(0.71–1.14)	1.16(0.95–1.40)	0.83(0.66–1.05)
Cancer	1.64(1.29–2.09)	1.33(1.01–1.77)	1.47(1.19–1.81)	1.28(1.00–1.63)	1.47(1.19–1.81)	1.17(0.93–1.49)
Ischaemic heart disease	1.34(1.14–1.58)	0.88(0.72–1.09)	1.30(1.13–1.49)	0.95(0.79–1.13)	1.71(1.51–1.96)	0.96(0.81–1.14)

*Note*: LLN is defined by normal values in a Danish population.^17^

^a^ Cumulative smoking (pack‐years) was excluded from the multivariable models, due to missing values for all former smokers.

^b^ Includes chronic obstructive pulmonary disease (COPD), chronic bronchitis, hyperinflated lungs and emphysema.

Table S3 shows additional spirometry results by sex for all participants aged 20 years and above. For men (*n* = 5984), FEV_1_ decreased from an average of 4.39 L for individuals aged 20 years to 2.29 L for individuals aged 80 + years (Table S3A). For women (*n* = 7015), it decreased from 3.24 L for people aged 20 years to 1.61 L for people aged 80 years (Table S3B). Across all ages, men had higher FEV_1_ than women. For men, the FEV_1_/FVC < 70% was 11.7 percentage points and 16.5 percentage points higher than FEV_1_/FVC < LLN 2.5% within age groups 70–79 and 80 + years, respectively. For women, the difference was 17.3 and 29.9 percentage points, respectively, in the two oldest age groups. The proportion of participants aged 20 and above with airway obstruction by the FEV_1_/FVC < LLN 5%, FEV_1_/FVC < LLN 2.5% and FEV1/FVC < 70% criteria in different subgroups of participants is shown in Tables S4A (men) and S4B (women).

## 4 DISCUSSION

Among LOFUS participants aged 35 years or older, 19% had airway obstruction judged by the fixed ratio criterion of FEV_1_/FVC < 70% or the LLN‐5% cut‐off. Using LLN2.5%, the proportion with airway obstruction was 12.2% overall. While some subgroups had similar proportions with FEV_1_/FVC below 70% and below LLN 2.5%, in other subgroups, the proportions were quite different, which may be due to different age composition of the groups.

A previous Danish study from Copenhagen estimated COPD prevalence in subjects aged over 35 years to be 17.4% based on FEV_1_/FVC < 70%.^7^ Another Danish study found prevalence of airway obstruction 18.0% using FEV_1_/FVC < 70% and 5.6% using LLN 2.5%.^8^

In a German cohort, the FEV_1_/FVC < 70% criterion was also shown to identify more individuals than LLN 5%, especially in older participants.^19^ The study also showed that the proportion of participants reporting that they had a physician diagnosis of COPD or took lung medication was higher among those who had airway obstruction by the LLN 5% criterion than among those with the FEV_1_/FVC < 70% criterion, with difference increasing with age. The use of the LLN criterion is arguably better, because the FEV_1_/FVC declines with age, irrespective of the presence of lung disease.^9^ In addition, using LLN, where each participant's spirometry result was compared to the expected value for that participant's age, height and sex, allows for comparison of proportions with airway obstruction across subgroups with different age composition.

Our study showed that fewer years in school were related to higher prevalence of airway obstruction (Table 1), while in multivariable analysis (Table 2), there was no significant association. It has been found previously that persons with shorter educational attainment have higher risk of developing COPD, and they also tend to have more severe disease, and higher risk for exacerbations and death, even when controlling for disease severity.^20^ In light of our results, this may be related to uneven distribution of risk factors and not educational level per se.

More intense physical activity was associated with lower proportion of individuals with airway obstruction. Previous studies suggested that regular physical activity reduces risk of COPD exacerbations, and among smokers, physical activity slowed lung function decline.^21^ In contrast, a Canadian cohort study found large waist circumference to be a strong predictor for impaired lung function with physical activity acting as a confounder.^22^ Unlike waist circumference, proportion with airway obstruction varied with BMI. COPD may be accompanied by weight loss and loss of muscle mass due to systemic involvement in some patients, resulting in low BMI. On the other hand, studies have also found higher prevalence of obesity among COPD patients,^7^ and in obese individuals, FEV_1_/FVC ratio may remain normal, and airway obstruction may be underdiagnosed.^23^

16.5% of participants reported daily smoking. This is lower than the figures for the latest national health survey, where 21% and 22.8%, respectively, of the population of the two municipalities in Lolland‐Falster reported daily smoking.^13^ Participants in LOFUS, as in other population health studies may be healthier and have higher socio‐economic status than the background population, leading to underestimation of disease burden.^24^

Strengths of the study include a large sample size, high proportion of participants completing spirometry and comprehensive data collection. All data on comorbidities, medication and physical activity were self‐reported and could potentially suffer from reporting bias. For example, most participants reporting other respiratory disease also reported asthma. Although asthma and COPD frequently coexist,^25^ we cannot be sure whether participants distinguish reliably between these conditions. Spirometry was performed without bronchodilator, which in a US study was associated with 50% higher prevalence of airway obstruction than post‐bronchodilator spirometry.^26^ Information on medication use on the day of spirometry was not collected. Neither was information on symptoms and therefore the estimates of airway obstruction must be interpreted carefully. Nevertheless, the study showed proportions of participants with airway obstruction in agreement with previous Danish studies.^7^, ^8^ A possible limitation was the use of 35 years, while many studies use 40 years, which may affect comparability. However, one of our aims was to relate the LOFUS data to a previous Danish study using 35 years as cut‐off.^7^ Table 1 shows that the age group 35–39 had the lowest prevalence of airway obstruction. Thus, calculating the AO prevalence in the 40+ population showed proportions of 19.8%, 19.3% and 12.4, using FEV_1_/FVC < 70, LLN 2.5 and LLN 5.0, respectively.

Knowing the prevalence of COPD—and undiagnosed COPD—in a population is important for estimating the potential for prevention, early diagnosis and treatment and for planning services. Data from Copenhagen showed that only a minority of people meeting the criteria for COPD received treatment.^7^ Among LOFUS participants who reported no known respiratory disease, 8.7% were found to have airway obstruction by the LLN 2.5% criterion, and 15.2% by the LLN 5% criterion (data not shown). For current daily smokers, these figures were 19.5% and 29.5%, respectively. Although no data on symptoms were available for assessment of clinical diagnoses, these numbers may give an indication of the extent of undiagnosed obstructive pulmonary disease.

The prevalence estimate depends on the spirometry criterion used.^27^ Even when using the LLN 5%, only 58.5% of men and 52.1% of women who reported respiratory disease had signs of airway obstruction. Consequently, a sizeable proportion of participants with self‐reported disease had spirometry result in the normal range. Whether this is due to error in self‐reported diagnosis, to effect of treatment of existing disease, or a combination of both, cannot be determined from the study. Nevertheless, it suggests that prevalence of lung disease may be underestimated when estimated from spirometry results in population studies such as LOFUS. Conversely, a Dutch study showed that population prevalence of COPD may be underestimated if including only self‐reported or physician diagnosed COPD, as substantial number of cases go undiagnosed.^28^

In a clinical setting, the spirometry result is interpreted in light of patient history and response to bronchodilator treatment. Such information was not available in the LOFUS database, and therefore, using the same cut‐off as in clinical diagnosis but without the clinical information may lead to overestimation of the COPD prevalence. In older age groups, the fixed ratio criterion (FEV_1_/FVC < 70%) may overestimate COPD prevalence even more than in younger age groups. The LLN criterion seems better suited for population studies,^25^ especially in older participants, and this has been found across geographical locations.^28^, ^29^, ^30^, ^31^ It has been suggested that LLN 2.5% cut‐off is more relevant in population studies than 5%.^8^ In future population studies, inclusion of clinical information and response to bronchodilator treatment would enable researchers to evaluate further which criterion gives the better estimate.

## 5 CONCLUSION

This was a descriptive cross‐sectional study presenting data on proportions with signs of airway obstruction in broad subgroups of participants in a population study from a rural part of Denmark using different cut‐offs for definition of airway obstruction. Our study shows that, using the same criteria to define airway obstruction as previous Danish studies, the population of Lolland‐Falster has comparable proportion airway obstruction, and hence possible COPD, 19% in participants aged 35 years or older. Our study also highlights how choosing a different cut‐off influences the estimate: using the LLN 2.5% cut‐off, which may be preferable for population studies, prevalence of airway obstruction was considerably lower at 12.2%. In addition, choice of criterion—LLN or FEV_1_/FVC ratio—influences the estimated prevalence of airway obstruction, especially with increasing age.

## CONFLICT OF INTEREST

All authors have no conflict of interest to declare.

## ETHICS STATEMENT

Informed written consent was obtained from all LOFUS participants. The LOFUS study was approved by the Region Zealand's Ethical Committee on Health Research (SJ‐421) and the Danish Data Protection Agency (REG‐024‐2015). LOFUS is registered in Clinical Trials (NCT02482896).

## AUTHOR CONTRIBUTIONS

Katja Kemp Jacobsen (KKJ), Randi Jepsen (RJ), Uffe Bodtger (UB), Knud Rasmussen (KR) and Gry St‐Martin (GSM) were involved in study design. RJ was involved in data collection. KKJ, RJ and GSM were involved with data analysis. RJ was involved with data curation. KKJ, RJ and GSM were involved with writing the original draft. KKJ, RJ, UB, KR and GSM were involved with writing—review and editing. All authors read and approved the final manuscript.

## Supporting information


**Table S1.** Characteristics of participants with and without spirometry results among 16 123 individuals aged ≥18 years in the LOFUS study.
**Table S2.** Characteristics of participants with successful spirometry and self‐reported other respiratory disease (Includes chronic obstructive pulmonary disease (COPD), chronic bronchitis, hyperinflated lungs and emphysema) among 1028 individuals aged ≥18 years in the LOFUS study.
**Table S3a**. Values for FEV_1_ (L) and FVC(L) (means (SD), proportion with FEV_1_ and FVC < 2.5 and 5% LLN and proportion with FEV_1_/FVC < 70% among 5984 men aged ≥20 years with successful spirometry in the LOFUS study according to age. LLN is defined by normal values in a Danish population (18).
**Table S3b**. Values for FEV_1_ (L) and FVC(L) (means (SD), proportion with FEV_1_ and FVC < 2.5 and 5% LLN and proportion with FEV_1_/FVC < 70% among 7015 women aged ≥20 years with successful spirometry in the LOFUS study according to age. LLN is defined by normal values in a Danish population (18).
**Table S4a**. Proportion with FEV_1_, FVC and FEV_1_/FVC < 2.5 and 5% LLN and proportion with FEV_1_/FVC < 70% among 5984 men ≥20 years with successful spirometry in the LOFUS study according to sex and other characteristics. LLN is defined by normal values in a Danish population (18).
**Table S4b**. Proportion with FEV_1_, FVC and FEV_1_/FVC < 2.5 and 5% LLN and proportion with FEV_1_/FVC < 70% among 7015 women aged ≥20 years with successful spirometry in the LOFUS study according to sex and characteristics. LLN is defined by normal values in a Danish population (18)

## Data Availability

Data are available upon reasonable request. Data from the study can be made available via Region Sjaelland following the Danish Data Protection Regulation.
