# Supplementary material for: Lung function in Lolland‐Falster Health Study (LOFUS)
Source: Clin Respir J. 2022 Sep 2;16(10):657–68. doi: 10.1111/crj.13536 (PMC9527155; doi:10.1111/crj.13536)
Supplement: Supplementary file 1 — Table S1. Characteristics of participants with and without spirometry results among 16 123 individuals aged ≥18 years in the LOFUS study. Table S2. Characteristics of participants with successful spirometry and self‐reported other respiratory disease (Includes chronic obstructive pulmonary disease (COPD), chronic bronchitis, hyperinflated lungs and emphysema) among 1028 individuals aged ≥18 years in the LOFUS study. Table S3a. Values for FEV1 (L) and FVC(L) (means (SD), proportion with FEV1 and FVC < 2.5 and 5% LLN and proportion with FEV1/FVC < 70% among 5984 men aged ≥20 years with successful spirometry in the LOFUS study according to age. LLN is defined by normal values in a Danish population (18). Table S3b. Values for FEV1 (L) and FVC(L) (means (SD), proportion with FEV1 and FVC < 2.5 and 5% LLN and proportion with FEV1/FVC < 70% among 7015 women aged ≥20 years with successful spirometry in the LOFUS study according to age. LLN is defined by normal values in a Danish population (18). Table S4a. Proportion with FEV1, FVC and FEV1/FVC < 2.5 and 5% LLN and proportion with FEV1/FVC < 70% among 5984 men ≥20 years with successful spirometry in the LOFUS study according to sex and other characteristics. LLN is defined by normal values in a Danish population (18). Table S4b. Proportion with FEV1, FVC and FEV1/FVC < 2.5 and 5% LLN and proportion with FEV1/FVC < 70% among 7015 women aged ≥20 years with successful spirometry in the LOFUS study according to sex and characteristics. LLN is defined by normal values in a Danish population (18) [file CRJ-16-657-s001.docx]

**Supplementary tables S1-S4**

S1 Table. Characteristics of participants with and without spirometry results among 16,123 individuals aged ≥18 years in the LOFUS study.

S2 Table. Characteristics of participants with successful spirometry and self-reported other respiratory disease (Includes chronic obstructive pulmonary disease (COPD), chronic bronchitis, hyperinflated lungs and emphysema) among 1,028 individuals aged ≥18 years in the LOFUS study.

S3a Table. Values for FEV_1_ (L) and FVC(L) (means (SD), proportion with FEV_1_ and FVC <2.5 and 5% LLN and proportion with FEV_1_/FVC < 70% among 5,984 men aged ≥20 years with successful spirometry in the LOFUS study according to age. LLN is defined by normal values in a Danish population (18).

S3b Table. Values for FEV_1_ (L) and FVC(L) (means (SD), proportion with FEV_1_ and FVC <2.5 and 5% LLN and proportion with FEV_1_/FVC < 70% among 7,015 women aged ≥20 years with successful spirometry in the LOFUS study according to age. LLN is defined by normal values in a Danish population (18).

S4a Table. Proportion with FEV_1_, FVC and FEV_1_/FVC <2.5 and 5% LLN and proportion with FEV_1_/FVC <70% among 5,984 men ≥20 years with successful spirometry in the LOFUS study according to sex and other characteristics. LLN is defined by normal values in a Danish population (18).

S4b Table. Proportion with FEV_1_, FVC and FEV_1_/FVC <2.5 and 5% LLN and proportion with FEV_1_/FVC <70% among 7,015 women aged ≥20 years with successful spirometry in the LOFUS study according to sex and characteristics. LLN is defined by normal values in a Danish population (18).

S1 Table. Characteristics of participants with and without spirometry results among 16,123 individuals aged ≥18 years in the LOFUS study.

|  | **Spirometry** | | | **No spirometry** | | |  |
| --- | --- | --- | --- | --- | --- | --- | --- |
|  | Men  n (%) | Women  n (%) | Total  n (%) | Men  n (%) | Women  n (%) | Total  n (%) | p-value |
| Total number (n) | 6,093(45.8) | 7,222(54.2) | 13,315 | 1,475(52.5) | 1,333(47.5) | 2,808 | 0.000 |
| **Age (years)** |  |  |  |  |  |  |  |
| Median (IQ range) | 59.0(46.5-69.1) | 57.4(45.2-67.7) | 58.1(45.7-68.4) | 61.9(49.6-71.2) | 60,6(46.2-70.4) | 61.2(47.9-70.8) | 0.0001 |
| <20 | 108(1.8) | 134(1.9) | 242(1.8) | 40(2.7) | 49(3.7) | 88(3.2) |  |
| 20-29 | 356(5.8) | 424(5.9) | 780(5.9) | 81(5.5) | 93(7.0) | 174(6.2) |  |
| 30-39 | 533(8.8) | 669(9.3) | 1,202(9.0) | 100(6.7) | 95(7.1) | 195(6.9) |  |
| 40-49 | 903(14.8) | 1,175(16.3) | 2,078(15.6) | 158(10.7) | 167(12.5) | 325(11.5) |  |
| 50-59 | 1,282(21.0) | 1,682(23.3) | 2.964(22.3) | 308(20.8) | 252(18.8) | 556(19.8) |  |
| 60-69 | 1,514(24.9) | 1,722(23.8) | 3,236(24.3) | 375(25.3) | 331(24.7) | 702(25.0) |  |
| 70-79 | 1,130(18.6) | 1,165(16.1) | 2,295(17.2) | 318(21.4) | 265(19.8) | 581(20.7) |  |
| 80+ | 267(4.4) | 251(3.5) | 518(3.9) | 103(7.2) | 84(6.5) | 187(6.9) | 0.000 |
| **Height (cm)** |  |  |  |  |  |  |  |
| Median (IQ range) | 178(173-183) | 165(161-169) | 171(164-177) | 178(173-182) | 164(160-169) | 171.5(164-178) | 0.0014 |
| <150 | 1(0.0) | 73(1.0) | 74(0.6) | 0(0.0) | 26(2.0) | 26(0.9) |  |
| 150-159 | 11(0.2) | 1,341(18.6) | 1,352(10.2) | 4(0.3) | 289(21.7) | 293(10.4) |  |
| 160-169 | 658(10.8) | 4,093(56.7) | 4,751(35.7) | 172(11.7) | 727(54.5) | 899(32.0) |  |
| 170-179 | 2,993(49.1) | 1,631(22.6) | 4,624(34.7) | 697(47.3) | 274(20.6) | 971(34.6) |  |
| 180-189 | 2,134(34.8) | 83(1.2) | 2,217(16.7) | 525(35.6) | 17(1.3) | 542(19.3) |  |
| 190-199 | 287(4.7) | 1(0.0) | 288(2.2) | 73(5.0) | 0(0.0) | 73(2.6) |  |
| 200+ | 9(0.1) | 0(0.0) | 9(0.1) | 4(0.3) | 0(0.0) | 4(0.2) | 0.000 |
| **BMI (kg/m^2^)** |  |  |  |  |  |  |  |
| Median (IQ range) | 27.0(24.5-30.1) | 25.8(22.9-29.9) | 26.5(23.7-30.0) | 27.4(24.7-30.6) | 26.3(22.9-30.3) | 26.9(24.0-30.4) | 0.0003 |
| BMI<18.5 | 37(0.6) | 124(1.8) | 161(1.2) | 9(0.6) | 32(2.5) | 41(1.5) |  |
| BMI 18.5-25 | 1,707(28.6) | 2,907(41.1) | 4,614(35.4) | 374(26.1) | 490(38.3) | 864(31.9) |  |
| BMI 25.1-30 | 2,704(45.2) | 2,294(32.4) | 4,998(38.3) | 638(44.5) | 412(32.2) | 1,050(38.7) |  |
| BMI>30 | 1,530(25.6) | 1,751(24.8) | 3,281(25.1) | 414(28.9) | 344(26.9) | 758(27.9) | 0.001 |
| Missing BMI | 115 | 146 | 261 | 40 | 55 | 95 |  |
| **Waist circumference (cm)** |  |  |  |  |  |  |  |
| Median (IQ range) | 100.0(92.0-109.0) | 88.0(79.0-98.0) | 94.0(84.0-104.0) | 102.0(94.0-110.0) | 88.0(79.0-100.0) | 97.0(85.0-106.0) | 0.0001 |
| < 94cm/80cm | 1,727(28.5) | 1,824(25.5) | 3,3551(26.9) | 353(24.3) | 344(26.1) | 697(25.2) |  |
| ≥ 94cm/80cm | 4,328(71.5) | 5,327(74.5) | 9,655(73.1) | 1,101(75.7) | 973(73.9) | 2,074(74.9) | 0.060 |
| Missing waist circumference | 38 | 71 | 109 | 21 | 16 | 37 |  |
| **School education** |  |  |  |  |  |  |  |
| ≤7 years | 675(11.9) | 525(7.7) | 1,200(9.6) | 209(15.3) | 135(11.0) | 344(13.3) |  |
| 8-9 years | 1,452(25.5) | 1,044(15.2) | 2,496(19.9) | 371(27.2) | 218(17.7) | 589(22.7) |  |
| 10-11 years | 1,880(33.0) | 2,264(33.1) | 4,144(33.0) | 417(33.9) | 417(30.6) | 834(32.2) |  |
| Graduated high school | 1,259(22.1) | 2,399(35.1) | 3,658(29.2) | 257(18.8) | 342(27.8) | 599(23.1) |  |
| Under education | 43(0.7) | 73(1.1) | 116(0.9) | 14(1.0) | 23(1.9) | 37(1.4) |  |
| Other | 383(6.7) | 546(8.0) | 929(7.4) | 97(7.1) | 94(7.7) | 191(7.4) | 0.000 |
| Missing school education | 401 | 371 | 772 | 110 | 104 | 214 |  |
| **Vocational education** |  |  |  |  |  |  |  |
| No education except from primary school | 578(10.1) | 927(13.5) | 1,505(12.0) | 161(11.8) | 241(19.6) | 402(15.5) |  |
| Smaller courses (e.g. semiskilled worker) | 402(7.1) | 322(4.7) | 724(5.8) | 113(8.3) | 86(7.0) | 199(7.7) |  |
| Vocational training | 2,603(45.7) | 2,617(38.2) | 5,220(41.6) | 594(43.4) | 448(36.3) | 1,042(40.1) |  |
| Short higher education | 522(9.2) | 556(8.1) | 1,078(8.6) | 155(11.6) | 84(7.0) | 239(9.5) |  |
| Middle higher education | 939(16.5) | 1,860(27.1) | 2,799(22.3) | 192(14.4) | 272(22.7) | 464(18.4) |  |
| Long higher education | 296(5.2) | 262(3.8) | 558(4.4) | 68(5.0) | 38(3.1) | 106(4.1) |  |
| Other | 362(6.4) | 315(4.6) | 677(5.4) | 85(6.2) | 64(5.2) | 149(5.7) | 0.000 |
| Missing vocational education | 391 | 363 | 754 | 107 | 100 | 207 |  |
| **Physical activity last year** |  |  |  |  |  |  |  |
| Sedentary activity | 672(11.8) | 802(11.7) | 1,474(11.7) | 186(13.6) | 184(14.8) | 370(14.2) |  |
| Moderate activity | 3,105(54.6) | 4,596(66.8) | 7,701(61.3) | 736(53.9) | 825(66.5) | 1,561(59.9) |  |
| Heavy activity | 1,708(30.0) | 1,385(20.1) | 3,093(24.6) | 393(28.8) | 216(17.4) | 609(23.4) |  |
| Heavy activity, at competition level | 201(3.5) | 98(1.4) | 299(2.4) | 50(3.7) | 16(1.3) | 66(2.5) |  |
| Missing physical activity | 407 | 341 | 748 | 110 | 92 | 202 | 0.004 |
| **Smoking** |  |  |  |  |  |  |  |
| Current daily smokers | 955(16.7) | 1,124(16.3) | 2,079(16.5) | 222(16.1) | 192(15.4) | 414(15.8) |  |
| Current, sometimes smokers | 206(3.6) | 168(2.4) | 374(3.0) | 52(3.8) | 22(1.8) | 74(2.8) |  |
| Former-smokers | 2,160(37.8) | 2,292(33.2) | 4,452(35.3) | 517(37.6) | 359(28.9) | 876(33.4) |  |
| Never-smokers | 2,397(41.9) | 3,313(48.0) | 5,710(45.3) | 586(42.6) | 671(53.9) | 1,257(48.0) | 0.094 |
| Missing smoking | 375 | 325 | 700 | 98 | 89 | 187 |  |
| **Cumulative smoking (pack-years)** | 20.0(6.6-36.8) | 18.6(8.0-29.5) | 19.1(7.1-33.0) | 24.3(6.9-40.4) | 20.7(9.9-32.5) | 21.9(7.6-36.3) | 0.0135 |
| >0-20 | 525(9.4) | 619(9.1) | 1,144(9.2) | 108(8.0) | 96(7.8) | 204(7.9) |  |
| 20-40 | 306(5.5) | 417(6.2) | 723(5.9) | 74(5.5) | 78(6.3) | 152(5.9) |  |
| 40-60 | 153(2.7) | 115(1.7) | 268(2.2) | 42(3.1) | 20(1.6) | 62(2.4) |  |
| >60 | 64(1.2) | 24(0.4) | 88(0.7) | 21(1.6) | 6(0.5) | 27(1.1) | 0.058 |
| Missing pack-years | 105 | 105 | 210 | 29 | 14 | 43 |  |
| **Chronic diseases** |  |  |  |  |  |  |  |
| Asthma | 402(6.6) | 590(8.2) | 992(7.5) | 74(5.0) | 89(6.7) | 163(5.8) | 0.002 |
| Other respiratory disease* | 448(7.4) | 580(8.0) | 1,028(7.7) | 97(6.6) | 87(6.5) | 184(6.6) | 0.002 |
| Allergy | 919(15.1) | 1,406(19.5) | 2,325(17.5) | 190(12.9) | 234(17.6) | 424(15.1) | 0.008 |
| Hypertension | 1,787(29.3) | 1,783(24.7) | 3,571(26.8) | 446(31.1) | 376(29.3) | 822(30.2) | 0.000 |
| Diabetes | 405(6.8) | 267(3.7) | 672(5.1) | 119(8.1) | 71(5.3) | 190(6.8) | 0.000 |
| Cancer | 247(4.1) | 237(3.3) | 484(3.6) | 51(4.0) | 67(4.7) | 118(4.3) | 0.150 |
| Ischaemic heart disease | 792(13.0) | 548(7.6) | 1,340(10.1) | 221(15.4) | 126(9.8) | 347(12.8) | 0.000 |

BMI: Body Mass Index.

* Includes chronic obstructive pulmonary disease (COPD), chronic bronchitis, hyperinflated lungs and emphysema.

S2 Table. Characteristics of participants with successful spirometry and self-reported other respiratory disease (Includes chronic obstructive pulmonary disease (COPD), chronic bronchitis, hyperinflated lungs and emphysema) among 1,028 individuals aged ≥18 years in the LOFUS study.

|  | **Respiratory disease** | | |
| --- | --- | --- | --- |
|  | Men  n (%) | Women  n (%) | Total  n (%) |
| Total number (n) | 448(43.6) | 580(56.4) | 1,028 |
| **Age (years)** |  |  |  |
| Median (IQ range) | 61.9(49.5-71.2) | 60.6(46.2-70.3) | 61.2(47.9-70.8) |
| ≤20 | 5(1.1) | 7(1.2) | 12(1.2) |
| 20-29 | 14(3.1) | 14(3.2) | 35(3.4) |
| 30-39 | 16(3.6) | 16(3.6) | 38(3.7) |
| 40-49 | 25(5.6) | 25(5.6) | 81(7.9) |
| 50-59 | 79(17.8) | 79(17.8) | 212(20.6) |
| 60-69 | 150(33.9) | 150(33.9) | 327(31.8) |
| 70-79 | 126(28.4) | 126(28.4) | 261(25.4) |
| 80+ | 33(7.5) | 33(7.5) | 62(6.0) |
| **Height (cm)** | 176.0(172.0-181.0) | 164.0(159.0-169.0) | 169.0(162.0-176.0) |
| <150 | 0(0.0) | 12(2.1) | 12(1.2) |
| 150-159 | 1(0.2) | 141(24.6) | 142(14.0) |
| 160-169 | 64(14.3) | 305(52.6) | 369(35.9) |
| 170-179 | 240(53.6) | 115(19.8) | 355(34.5) |
| 180-189 | 128(28.6) | 7(1.2) | 135(13.1) |
| 190-199 | 15(3.4) | 0(0.0) | 15(1.5) |
| 200+ | 0(0.0) | 0(0.0) | 0(0.0) |
| **BMI (kg/m^2^)** |  |  |  |
| Median (IQ range) | 27.2(24.5-30.9) | 26.6(23.3-30.8) | 26.9(23.8-30.8) |
| BMI<18.5 | 6(1.4) | 15(2.6) | 21(2.1) |
| BMI 18.5-25 | 120(28.0) | 205(36.1) | 325(32.6) |
| BMI 25.1-30 | 174(40.6) | 189(33.3) | 363(36.4) |
| BMI>30 | 129(30.1) | 159(28.0) | 288(28.9) |
| Missing BMI | 19(4.2) | 12(2.1) | 31(3.0) |
| **Waist circumference (cm)** |  |  |  |
| Median (IQ range) | 104(95.0-102.0) | 91.0(81.0-102.0) | 97.0(87.0-108.0) |
| < 94cm/80cm | 90(20.6) | 107(18.6) | 197(19.5) |
| ≥ 94cm/80cm | 348(79.5) | 467(81.4) | 815(80.5) |
| Missing waist circumference | 10(2.2) | 6(1.0) | 16(0.6) |
| **School education** |  |  |  |
| ≤7 years | 96(21.7) | 70(12.1) | 166(16.3) |
| 8-9 years | 148(33.4) | 140(24.3) | 288(28.2) |
| 10-11 years | 109(24.6) | 185(32.1) | 294(28.8) |
| Graduated high school | 62(14.0) | 129(22.4) | 191(18.7) |
| Under education | 2(0.5) | 4(0.7) | 6(0.6) |
| Other | 26(5.9) | 49(8.5) | 75(7.4) |
| Missing school education | 5(1.1) | 3(0.5) | 8(0.8) |
| **Vocational education** |  |  |  |
| No education except from primary school | 51(11.5) | 98(17.1) | 149(14.7) |
| Smaller courses (e.g. semiskilled worker) | 41(9.3) | 45(7.9) | 86(8.5) |
| Vocational training | 203(45.9) | 235(41.0) | 438(43.2) |
| Short higher education | 23(5.3) | 34(6.0) | 57(5.6) |
| Middle higher education | 61(13.8) | 116(20.2) | 177(17.4) |
| Long higher education | 22(5.0) | 14(2.4) | 36(3.6) |
| Other | 41(9.3) | 31(5.4) | 72(7.1) |
| Missing vocational education | 6(1.3) | 7(1.2) | 13(0.3) |
| **Physical activity last year** |  |  |  |
| Sedentary activity | 84(19.1) | 118(20.5) | 202(19.9) |
| Moderate activity | 253(57.5) | 373(64.9) | 626(61.7) |
| Heavy activity | 92(20.9) | 78(13.6) | 170(16.8) |
| Heavy activity, at competition level | 11(2.5) | 6(1.0) | 17(1.7) |
| Missing physical activity | 8(1.8) | 5(0.9) | 13(1.3) |
| **Smoking** |  |  |  |
| Current daily smokers | 123(27.7) | 156(27.0) | 279(27.3) |
| Current, sometimes smokers | 18(4.1) | 17(3.0) | 35(3.4) |
| Former-smokers | 210(47.3) | 243(42.1) | 453(44.4) |
| Never-smokers | 93(21.0) | 161(27.9) | 254(24.9) |
| Missing smoking | 4(0.9) | 3(0.5) | 7(0.7) |
| **Cumulative smoking (pack-years)** |  |  |  |
| >0-20 | 56(12.8) | 58(10.3) | 114(11.4) |
| 20-40 | 38(8.7) | 60(10.6) | 98(9.8) |
| 40-60 | 22(5.0) | 31(5.5) | 53(5.3) |
| >60 | 18(4.1) | 11(2.0) | 29(2.9) |
| Missing pack-years | 7(5.0) | 13(7.5) | 20(6.4) |
| **Chronic diseases** |  |  |  |
| Asthma | 299(66.7) | 473(81.6) | 772(75.1) |
| Allergy | 146(32.6) | 240(41.4) | 386(37.6) |
| Hypertension | 200(44.6) | 216(37.2) | 416(40.5) |
| Diabetes | 53(11.8) | 41(7.1) | 94(9.1) |
| Cancer | 32(7.1) | 32(5.5) | 64(6.2) |
| Ischaemic heart disease | 117(26.1) | 94(16.2) | 211(20.5) |

BMI: Body Mass Index.

S3a Table. Values for FEV_1_ (L) and FVC(L) (means (SD), proportion with FEV_1_ and FVC <2.5 and 5% LLN and proportion with FEV_1_/FVC < 70% among 5,984 men aged ≥20 years with successful spirometry in the LOFUS study according to age. LLN is defined by normal values in a Danish population (18).

| **Men** | | | | | | | | | | | |
| --- | --- | --- | --- | --- | --- | --- | --- | --- | --- | --- | --- |
|  | **Number**  **(%)** | **FEV_1_,**  **L**  **Mean (SD)** | **FEV_1_**  **<LLN 5% (%)** | **FEV_1_**  **<LLN 2.5%**  **(%)** | **FVC,**  **L**  **Mean (SD)** | **FVC**  **< LLN 5%**  **(%)** | **FVC**  **<LLN 2.5%**  **(%)** | **FEV_1_/FVC**  **(%)**  **Mean (SD)** | **FEV_1_/FVC**  **<LLN 5% (%)** | **FEV_1_/FVC**  **<LLN 2.5%**  **(%)** | **FEV_1_/FVC**  **<70%**  **(%)** |
| Total values | 5,984 | 3.36(0.84) | 17.3 | 13.4 | 4.42(0.97) | 9.8 | 7.1 | 75.9(8.4) | 18.7 | 12.2 | 17.7 |
| **Age (years)** |  |  |  |  |  |  |  |  |  |  |  |
| 20-29 | 356 | 4.39(0.64) | 8.2 | 4.8 | 5.44(0.82) | 5.6 | 3.7 | 81.1(6.4) | 11.0 | 5.3 | 4.5 |
| 30-39 | 533 | 4.14(0.62) | 10.7 | 7.3 | 5.27(0.75) | 5.3 | 3.2 | 78.9(6.4) | 14.1 | 7.3 | 5.8 |
| 40-49 | 902 | 3.91(0.63) | 11.9 | 8.9 | 5.02(0.79) | 6.1 | 4.2 | 78.0(6.5) | 12.8 | 7.5 | 8.0 |
| 50-59 | 1,282 | 3.50(0.63) | 18.7 | 13.8 | 4.59(0.76) | 10.0 | 7.2 | 76.4(7.5) | 18.0 | 11.4 | 15.0 |
| 60-69 | 1,514 | 3.08(0.65) | 22.0 | 18.0 | 4.12(0.76) | 12.4 | 9.1 | 74.6(8.6) | 21.7 | 14.5 | 22.1 |
| 70-79 | 1,130 | 2.70(0.61) | 20.3 | 16.1 | 3.70(0.74) | 12.6 | 9.4 | 73.4(9.3) | 23.0 | 16.5 | 28.2 |
| 80+ | 267 | 2.29(0.57) | 15.7 | 12.7 | 3.21(0.68) | 9.4 | 7.9 | 71.6(10.2) | 25.8 | 19.1 | 35.6 |

FEV_1_: Forced Expiratory Volume in one second, FVC: Forced Expiratory Vital capacity. LLN: lower limit of normal. BMI: Body Mass Index.

* Includes chronic obstructive pulmonary disease (COPD), chronic bronchitis, hyperinflated lungs and emphysema.

S3b Table. Values for FEV_1_ (L) and FVC(L) (means (SD), proportion with FEV_1_ and FVC <2.5 and 5% LLN and proportion with FEV_1_/FVC < 70% among 7,015 women aged ≥20 years with successful spirometry in the LOFUS study according to age. LLN is defined by normal values in a Danish population (18).

| **Women** | | | | | | | | | | | |
| --- | --- | --- | --- | --- | --- | --- | --- | --- | --- | --- | --- |
|  | **Number**  **(%)** | **FEV_1_,**  **L**  **Mean (SD)** | **FEV_1_**  **<LLN 5%**  **(%)** | **FEV_1_**  **<LLN 2.5%**  **(%)** | **FVC,**  **L**  **Mean (SD)** | **FVC**  **<LLN 5%**  **(%)** | **FVC**  **<LLN 2.5%**  **(%)** | **FEV_1_/FVC**  **(%)**  **Mean (SD)** | **FEV_1_/FVC**  **<LLN 5%**  **(%)** | **FEV_1_/FVC**  **<LLN 2.5%**  **(%)** | **FEV_1_/FVC**  **<70%**  **(%)** |
| Total values | 7,015 | 2.49(0.63) | 16.1 | 12.0 | 3.25(0.70) | 8.7 | 6.1 | 76.2(8.3) | 17.9 | 11.0 | 17.3 |
| **Age (years)** |  |  |  |  |  |  |  |  |  |  |  |
| 20-29 | 422 | 3.24(0.43) | 9.5 | 5.7 | 3.88(0.53) | 9.5 | 5.7 | 83.8(6.4) | 8.3 | 3.8 | 2.4 |
| 30-39 | 666 | 3.09(0.44) | 11.4 | 7.8 | 3.85(0.54) | 6.0 | 4.1 | 80.4(6.2) | 14.6 | 8.1 | 5.9 |
| 40-49 | 1,169 | 2.89(0.45) | 14.5 | 10.4 | 3.69(0.55) | 5.8 | 4.1 | 78.2(6.3) | 16.1 | 9.0 | 8.3 |
| 50-59 | 1,672 | 2.54(0.46) | 18.8 | 13.2 | 3.35(0.55) | 9.7 | 6.3 | 76.1(7.2) | 19.5 | 11.2 | 14.4 |
| 60-69 | 1,705 | 2.23(0.49) | 19.1 | 15.3 | 3.00(0.55) | 10.4 | 7.3 | 74.0(8.6 | 20.6 | 14.0 | 22.9 |
| 70-79 | 1,150 | 1.94(0.43) | 15.0 | 12.1 | 2.66(0.51) | 9.0 | 6.9 | 73.2(9.0) | 19.1 | 12.9 | 30.2 |
| 80+ | 231 | 1.61(0.41) | 12.6 | 10.8 | 2.23(0.45) | 9.5 | 6.9 | 71.1(9.1) | 16.9 | 9.1 | 39.0 |

FEV_1_: Forced Expiratory Volume in one second, FVC: Forced Expiratory Vital capacity. LLN: lower limit of normal. BMI: Body Mass Index.

* Includes chronic obstructive pulmonary disease (COPD), chronic bronchitis, hyperinflated lungs and emphysema.

S4a Table. Proportion with FEV_1_, FVC and FEV_1_/FVC <2.5 and 5% LLN and proportion with FEV_1_/FVC <70% among 5,984 men ≥20 years with successful spirometry in the LOFUS study according to sex and other characteristics. LLN is defined by normal values in a Danish population (18).

| **Men**  **(%)** | | | | | | | | |
| --- | --- | --- | --- | --- | --- | --- | --- | --- |
|  | **Number**  **(%)** | **FEV_1_ <LLN 5%** | **FEV_1_**  **<LLN 2.5%** | **FVC**  **<LLN 5%** | **FVC**  **<LLN 2.5%** | **FEV_1_/FVC**  **<LLN 5%** | **FEV_1_/FVC**  **<LLN 2.5%** | **FEV_1_/FVC**  **<70%** |
| **BMI (kg/m^2^)** |  |  |  |  |  |  |  |  |
| BMI<18.5 | 32 | 37.5 | 34.4 | 15.6 | 6.3 | 40.6 | 31.3 | 32.4 |
| BMI 18.5-25 | 1,635 | 13.4 | 10.1 | 5.9 | 4.8 | 21.7 | 15.0 | 20.2 |
| BMI 25.1-30 | 2,683 | 14.9 | 11.6 | 7.4 | 5.3 | 17.5 | 11.3 | 16.4 |
| BMI>30 | 1,519 | 24.0 | 18.4 | 17.2 | 12.1 | 16.5 | 9.9 | 15.4 |
| **Waist circumference (cm)** |  |  |  |  |  |  |  |  |
| < 94cm/80cm | 1,643 | 11.4 | 8.3 | 4.6 | 3.3 | 19.1 | 12.8 | 16.3 |
| ≥ 94cm/80cm | 4,303 | 19.4 | 15.2 | 11.6 | 8.4 | 18.4 | 11.8 | 18.0 |
| **School education** |  |  |  |  |  |  |  |  |
| ≤7 years | 675 | 23.1 | 19.1 | 13.8 | 10.8 | 27.1 | 19.1 | 30.2 |
| 8-9 years | 1,445 | 20.4 | 16.5 | 12.5 | 9.3 | 20.1 | 13.3 | 19.7 |
| 10-11 years | 1,852 | 15.8 | 11.9 | 7.8 | 5.5 | 16.5 | 10.3 | 14.7 |
| Graduated high school | 1,213 | 12.2 | 8.8 | 7.2 | 4.6 | 15.8 | 8.8 | 13.6 |
| Under education | 15 | 6.7 | 6.7 | 6.7 | 6.7 | 13.3 | 13.3 | 9.3 |
| Other | 380 | 16.8 | 12.6 | 9.0 | 7.1 | 17.4 | 13.4 | 16.5 |
| **Vocational education** |  |  |  |  |  |  |  |  |
| No education except from primary school | 493 | 21.3 | 17.4 | 12.8 | 9.3 | 20.5 | 14.6 | 18.0 |
| Smaller courses (e.g. semiskilled worker) | 397 | 22.2 | 17.9 | 12.9 | 9.1 | 23.9 | 15.4 | 21.1 |
| Vocational training | 2,602 | 16.2 | 12.8 | 9.3 | 7.0 | 17.5 | 11.2 | 16.6 |
| Short higher education | 522 | 14.5 | 11.3 | 7.7 | 5.8 | 17.1 | 11.9 | 17.4 |
| Middle higher education | 939 | 14.9 | 11.0 | 8.7 | 5.9 | 17.7 | 10.4 | 17.0 |
| Long higher education | 296 | 13.2 | 9.1 | 6.4 | 4.1 | 19.9 | 12.5 | 18.9 |
| Other | 359 | 25.4 | 18.4 | 12.8 | 9.5 | 22.3 | 15.0 | 22.4 |
| **Physical activity last year** |  |  |  |  |  |  |  |  |
| Sedentary activity | 656 | 29.6 | 23.6 | 19.4 | 13.6 | 25.5 | 17.4 | 23.9 |
| Moderate activity | 3,077 | 17.4 | 14.1 | 9.9 | 7.3 | 18.7 | 12.2 | 18.3 |
| Heavy activity | 1,689 | 12.3 | 8.6 | 5.9 | 4.2 | 16.5 | 10.0 | 15.3 |
| Heavy activity, at competition level | 170 | 5.3 | 4.1 | 2.4 | 2.4 | 12.9 | 8.2 | 9.0 |
| **Smoking** |  |  |  |  |  |  |  |  |
| Current daily smokers | 946 | 30.0 | 24.7 | 13.5 | 10.3 | 33.8 | 24.1 | 31.1 |
| Current sometimes smokers | 194 | 16.0 | 9.3 | 7.7 | 6.7 | 22.7 | 11.9 | 17.5 |
| Former-smokers | 2,153 | 19.5 | 15.1 | 10.9 | 8.3 | 20.9 | 14.0 | 21.7 |
| Never-smokers | 2,331 | 9.8 | 7.2 | 7.1 | 4.5 | 10.2 | 5.4 | 8.9 |
| **Cumulative smoking (pack-years)** |  |  |  |  |  |  |  |  |
| >0-20 | 512 | 19.5 | 15.2 | 10.4 | 7.8 | 26.8 | 16.8 | 22.5 |
| 20-40 | 306 | 30.7 | 26.5 | 11.8 | 10.1 | 31.1 | 23.5 | 28.1 |
| 40-60 | 153 | 46.4 | 37.3 | 20.3 | 13.1 | 52.9 | 41.2 | 52.9 |
| >60 | 64 | 50.0 | 40.6 | 23.4 | 21.9 | 50.0 | 31.3 | 50.0 |
| **Chronic diseases** |  |  |  |  |  |  |  |  |
| Asthma | 396 | 47.0 | 42.9 | 22.2 | 18.4 | 51.0 | 40.9 | 47.5 |
| Other respiratory disease* | 443 | 56.4 | 51.5 | 26.0 | 21.2 | 58.5 | 48.8 | 58.0 |
| Allergy | 896 | 19.1 | 14.8 | 10.7 | 7.5 | 22.5 | 14.0 | 19.3 |
| Hypertension | 1,786 | 23.2 | 19.0 | 15.3 | 11.4 | 20.5 | 13.7 | 22.4 |
| Diabetes | 405 | 32.4 | 25.6 | 20.7 | 15.3 | 20.5 | 11.6 | 22.0 |
| Cancer | 246 | 24.0 | 19.5 | 16.3 | 11.4 | 24.8 | 18.3 | 28.5 |
| Ischaemic heart disease | 791 | 29.2 | 24.0 | 19.0 | 15.3 | 23.1 | 14.4 | 25.5 |

FEV_1_: Forced Expiratory Volume in one second, FVC: Forced Expiratory Vital capacity. LLN: lower limit of normal. BMI: Body Mass Index.

*Includes COPD, chronic bronchitis, and emphysema.

S4b Table. Proportion with FEV_1_, FVC and FEV_1_/FVC <2.5 and 5% LLN and proportion with FEV_1_/FVC <70% among 7,015 women aged ≥20 years with successful spirometry in the LOFUS study according to sex and characteristics. LLN is defined by normal values in a Danish population (18).

| **Women**  **(%)** | | | | | | | | |
| --- | --- | --- | --- | --- | --- | --- | --- | --- |
|  | **Number**  **(%)** | **FEV_1_ <LLN 5%** | **FEV_1_ <LLN 2.5%** | **FVC <LLN 5%** | **FVC <LLN 2.5%** | **FEV_1_/FVC**  **<LLN 5%** | **FEV_1_/FVC**  **<LLN 2.5%** | **FEV_1_/FVC**  **<70%** |
| **BMI (kg/m^2^)** |  |  |  |  |  |  |  |  |
| BMI<18.5 | 118 | 29.7 | 24.6 | 15.3 | 10.2 | 33.1 | 20.3 | 31.5 |
| BMI 18.5-25 | 2,789 | 13.4 | 10.0 | 6.6 | 4.5 | 20.2 | 12.4 | 19.0 |
| BMI 25.1-30 | 2,234 | 15.8 | 11.9 | 7.8 | 5.4 | 17.6 | 10.7 | 17.4 |
| BMI>30 | 1,732 | 19.8 | 14.6 | 12.6 | 8.7 | 13.5 | 8.5 | 12.6 |
| **Waist circumference (cm)** |  |  |  |  |  |  |  |  |
| <94cm/80cm | 1,712 | 12.5 | 8.9 | 6.6 | 4.6 | 18.9 | 11.0 | 15.7 |
| ≥94cm/80cm | 5,233 | 17.2 | 13.0 | 9.4 | 6.4 | 17.5 | 11.0 | 17.6 |
| **School education** |  |  |  |  |  |  |  |  |
| ≤7 years | 507 | 17.6 | 15.2 | 11.1 | 8.7 | 20.9 | 13.6 | 31.4 |
| 8-9 years | 1,018 | 23.0 | 18.7 | 12.2 | 9.0 | 22.1 | 15.1 | 25.7 |
| 10-11 years | 2,232 | 16.6 | 12.3 | 9.1 | 6.0 | 18.6 | 11.1 | 17.8 |
| Graduated high school | 2,335 | 11.1 | 7.8 | 5.5 | 3.9 | 15.9 | 8.8 | 11.5 |
| Under education | 40 | 22.5 | 15.0 | 20.0 | 10.0 | 10.0 | 5.0 | 2.7 |
| Other | 540 | 17.8 | 11.5 | 8.9 | 5.7 | 15.0 | 10.2 | 12.6 |
| **Vocational education** |  |  |  |  |  |  |  |  |
| No education except from primary school | 801 | 18.5 | 15.0 | 11.2 | 7.6 | 18.9 | 12.1 | 18.1 |
| Smaller courses (e.g. semiskilled worker) | 319 | 24.8 | 21.3 | 12.2 | 8.8 | 24.8 | 15.1 | 24.2 |
| Vocational training | 2,583 | 16.2 | 12.0 | 7.8 | 5.5 | 18.8 | 11.4 | 18.2 |
| Short higher education | 553 | 13.7 | 9.2 | 7.2 | 5.1 | 13.7 | 8.7 | 15.1 |
| Middle higher education | 1,854 | 12.8 | 9.0 | 7.1 | 5.1 | 16.3 | 9.4 | 14.1 |
| Long higher education | 261 | 11.5 | 7.3 | 6.9 | 4.6 | 18.4 | 10.7 | 13.7 |
| Other | 311 | 20.6 | 17.4 | 13.8 | 9.0 | 19.6 | 13.8 | 24.4 |
| **Physical activity last year** |  |  |  |  |  |  |  |  |
| Sedentary activity | 773 | 27.0 | 22.3 | 18.0 | 12.7 | 21.1 | 14.5 | 21.3 |
| Moderate activity | 4,505 | 15.7 | 11.6 | 7.9 | 5.4 | 18.3 | 11.2 | 17.7 |
| Heavy activity | 1,343 | 10.1 | 7.0 | 5.2 | 3.8 | 15.6 | 8.6 | 14.3 |
| Heavy activity, at competition level | 77 | 6.5 | 3.9 | 5.2 | 3.9 | 7.8 | 2.6 | 3.1 |
| **Smoking** |  |  |  |  |  |  |  |  |
| Current daily smokers | 1,100 | 29.1 | 23.4 | 13.3 | 10.0 | 34.0 | 23.7 | 29.0 |
| Current sometimes smokers | 148 | 22.3 | 14.2 | 10.8 | 9.5 | 24.3 | 15.5 | 17.9 |
| Former-smokers | 2,264 | 18.2 | 14.0 | 8.8 | 6.1 | 20.4 | 13.0 | 21.4 |
| Never-smokers | 3,203 | 9.2 | 6.3 | 6.5 | 4.2 | 10.5 | 5.0 | 10.4 |
| **Cumulative smoking (pack-years)** |  |  |  |  |  |  |  |  |
| >0-20 | 598 | 19.7 | 14.6 | 9.0 | 7.0 | 26.6 | 16.6 | 20.2 |
| 20-40 | 408 | 35.5 | 29.2 | 15.4 | 11.3 | 39.5 | 29.4 | 36.0 |
| 40-60 | 113 | 46.9 | 36.3 | 25.7 | 19.5 | 46.0 | 31.9 | 40.5 |
| >60 | 24 | 54.2 | 50.0 | 20.8 | 20.8 | 62.5 | 50.0 | 54.2 |
| **Chronic diseases** |  |  |  |  |  |  |  |  |
| Asthma | 572 | 45.5 | 38.1 | 19.0 | 15.7 | 46.5 | 40.9 | 43.7 |
| Other respiratory disease* | 561 | 51.3 | 43.5 | 23.5 | 19.3 | 52.1 | 42.1 | 52.4 |
| Allergy | 1,365 | 18.3 | 14.4 | 8.6 | 5.9 | 20.8 | 13.7 | 19.5 |
| Hypertension | 1,747 | 19.8 | 16.3 | 11.9 | 9.0 | 18.6 | 13.7 | 23.1 |
| Diabetes | 264 | 29.9 | 22.4 | 19.7 | 13.3 | 18.6 | 11.7 | 19.5 |
| Cancer | 234 | 24.8 | 21.8 | 15.8 | 10.3 | 26.1 | 18.4 | 29.0 |
| Ischaemic heart disease | 523 | 27.0 | 23.3 | 17.0 | 13.7 | 22.4 | 16.4 | 30.5 |

FEV_1_: Forced Expiratory Volume in one second, FVC: Forced Expiratory Vital capacity. LLN: lower limit of normal. BMI: Body Mass Index.

*Includes chronic obstructive pulmonary disease (COPD), chronic bronchitis, hyperinflated lungs and emphysema.
